# Risk Factors Associated with Emergency Department Recidivism in the Older Adult

**DOI:** 10.5811/westjem.2019.7.43073

**Published:** 2019-10-14

**Authors:** Sophia Sheikh

**Affiliations:** University of Florida–Jacksonville, Department of Emergency Medicine, Jacksonville, Florida

## Abstract

Our objective was to review risk factors predictive of older adult recidivism in the emergency department. Certain risk factors and themes commonly occurred in the literature. These recurring factors included increasing age, male gender, certain diagnoses (abdominal pain, traumatic injuries, and respiratory complaints), psychosocial factors (depression, anxiety, poor social support, and limited health literacy), and poor general health (cognitive health and physical functioning). Many of the identified risk factors are not easily modifiable posing a significant challenge in the quest to develop and implement effective intervention strategies.

## INTRODUCTION

Emergency department (ED) overutilization costs the U.S. healthcare system nearly $38 billion annually.^1^–^5^ ED recidivism (defined as ED returns after discharge from an index ED visit) by older adult patients is a substantial contributing factor to ED overutilization with estimated rates varying between nearly 20% to over 40%, depending on time elapsed since the index ED visit, 30 days to six months, respectively.^6^, ^7^ Older adults have more comorbid conditions and complex medical histories as compared to younger adults, often necessitating more expensive and lengthy ED diagnostic testing.^2^, ^8^, ^9^ Their utilization of the ED despite having health insurance and a primary care physician, suggests other contributors, such as poor health literacy, cognitive impairment, and lack of social support.^1^,^3^,^10^–^12^

Understanding the factors leading to ED recidivism in older adults is necessary to build prevention strategies to decrease unnecessary testing, overutilization of healthcare resources, and hospital admissions. High rates of recidivism coupled with the projected rise in the older adult population makes it critical that effective prevention strategies targeting older adults are developed.^13^ This narrative review will discuss risk factors for ED recidivism in older adults.

## METHODS

We searched the PubMed database using the terms *emergency department*, *recidivism* or *return(s), and older adults* from 1985 to November 1, 2018. We identified a total of 185 articles. Studies excluded were those with the following charcteristics: 1) did not include community-dwelling older adults (defined as patients over age 55); 2) did not provide a separate sub-analysis of the older adult patients; and 3) included patients with scheduled ED returns. Various time intervals appear in the literature, including 2, 3, 7, 14, and 30 or more days post-index visit. Studies looking at ED returns within 12 months of the index ED visit were included in this review to ensure that all potential contributing factors were explored. After applying the above exclusion criteria, we performed a review of the cited references for the remaining publications to capture additional pertinent literature. Table 1 displays the characteristics of the 19 included studies.

## RISK FACTORS REPORTED IN THE LITERATURE

### Demographics

Age increases the odds of returns in older adults.^6^, ^12^, ^14^ In a study of the general ED population within the United States (U.S.), patients over 65 years of age were three times more likely to return and be hospitalized within 72 hours of an ED visit compared to those under age 30. Additionally, risk for ED recidivism and hospitalization appears to increase with advanced age.^12^, ^15^ An increase in age of one year above the age of 70 was found to be an independent predictor for 30-day ED returns in Dutch older adults.^15^ However, this increased risk appears to decline after age 85.^14^ Male gender was also found to increase odds of returns in U.S., Dutch and Canadian older adult populations.^12^, ^15^, ^16^ Other demographic data, such as ethnicity and insurance status, were not predictive of returns by older adults.^10^, ^12^, ^16^

### ED Diagnoses and Pain

Several diagnoses in older adults are associated with ED returns (Table 2). Diagnoses most commonly reported as predictive of recidivism include those related to the respiratory system, traumatic injuries, and pain (particularly chest and abdominal pain). Respiratory diagnoses were found to be predictive not only of 30-day recidivism but of frequent recidivism (defined as three or more return visits; excluding index visit within six months).^6^ It is possible that the association of respiratory diagnoses with ED recidivism may reflect the season in which the studies were conducted. Information regarding the time of year the studies were conducted or whether a large percentage of the study population were enrolled in the fall and winter months is not available. Another possibility is that patients with respiratory diagnoses may have underlying chronic respiratory conditions such as emphysema or asthma and that these patients represent a sicker population.

Common ED complaints in older adults include abdominal and chest pain. According to the National Health Statistics Reports of 2007, abdominal pain was the third most common reason for ED visits among all adults aged 65 years or older.^19^ Many patients presenting to the ED with abdominal pain or chest pain often do not receive a definitive diagnosis for the cause of their complaint despite extensive diagnostic testing. While clinicians feel safe discharging a patient with negative test results, believing that testing did not reveal any cause for emergent treatment or admission, this news may produce the opposite effect in patients due to this diagnostic uncertainty and fear of the unknown cause of their complaints. This lack of diagnostic certainty may lead patients to return to the ED in the hope of finding an answer or out of fear if the symptoms return.^20^–^22^ The psychological component experienced by patients during their ED encounters is often overlooked and is a potential area of focus for study and improvement.

All types of pain appear to increase the odds of ED returns in older adults. Furthermore, pain complaints may be predictive of *frequent* returns (more than five visits in one year), particularly in those discharged from the ED with a prescription opioid.^23^–^25^ Patients discharged with prescription opioids who are properly educated on prescription opioid medications may be less likely to experience opioid-related adverse events, potentially minimizing ED recidivism.^23^

### Comorbid Conditions and Chronic Illness

The presence of certain comorbid conditions such as depression, heart disease, diabetes, stroke, and cancer also increase ED recidivism in older adults.^6^, ^25^ Poor mental health, depression, and diabetes were predictive not only of 30-day returns but of frequent returns.^6^, ^14^, ^26^ A history of psychiatric disorders is a common risk factor identified in several studies with one reporting it as predictive of frequent ED visits (more than five visits in one year).^24^, ^27^ In a study of low-income, homebound older adults with depression, a positive association was found between the Hamilton Rating Scale for Depression scores and frequency of ED visits.^25^ Non-cardiac, non-traumatic body pain was the most common reason for recidivism in this older adult population suffering from depression, highlighting the well-established link between depression and pain.

**Population Health Research Capsule**

What do we already know about this issue?

*ED overutilization is a significant burden on the healthcare system. ED recidivism by older adults is a substantial contributor to this overutilization*.

What was the research question?

*The objective of this report is to review risk factors predictive of older adult ED recidivism*.

What was the major finding of the study?

*Risk factors included age, male gender, certain diagnoses, and psychosocial factors. Many are not easily modifiable*.

How does this improve population health?

*Identifying risk factors and effective prevention strategies are essential given the expected population growth for this group*.

While the literature suggests that specific comorbid conditions are associated with increased recidivism, overall co-morbidity burden, as measured by the Charlson Comorbidity Index, is not. Although intuitively it would seem that patients with high co-morbidity burden would be more likely to return to the ED, La Mantia et al. found no association between Charlson comorbidity scores and ED recidivism.^10^ The presence of chronic illness in older adults returning, often frequently, to the ED suggests that at baseline these high-risk patients are sicker with a high burden of comorbidities requiring treatment with multiple medications. This likely explains the reporting of polypharmacy (taking three or more medications) as an independent predictor for 30-day ED returns in older adults.^6^, ^15^ Additionally, recent hospitalization, an indicator of clinical illness severity, was also found to be an independent predictive factor for repeat and frequent ED visits in older adults.^6^, ^28^

Reasons for returning to the ED in this older adult population suffering with chronic illness may stem from the following: seeking reassurance regarding their condition; noncompliance with treatment plans leading to complications; compliance with treatment plans but still developing complications from their condition; not understanding the course of their disease; or inadequate education regarding their discharge plan.

### Psychosocial Factors

Several psychosocial factors are associated with returns visits in older adults. These include lack of social support, marital status, and anxiety.^6^, ^28^ Divorced, separated, or widowed patients have more than double the increased odds for early returns within 30 days; conversely, patients who never married were significantly less likely to return.^6^ An explanation proposed by McCusker et al. for this finding is that patients who never married are more self-sufficient and independent than those who are currently or have previously been married. Reporting a perceived lack of social support by the patient was predictive of both 30-day and frequent returns (three or more within six months).^6^ Patients who are divorced, separated, or widowed may feel they have less social support than their married counterparts to assist in their healthcare needs.

Other psychosocial factors reported in the literature include anxiety and substance abuse such as daily alcohol use. Naughton et al. found a 13% increase in the risk of revisits per one unit increase in anxiety scores on the Hospital Anxiety and Depression Scale.^29^ The association between anxiety and ED recidivism supported by the literature is not surprising, particularly when a patient may not receive a definitive cause for their symptoms. Patients may experience fear and uncertainty regarding their health leading to anxiety.^20^, ^21^ This coupled with a perceived poor social support system may lead these patients to return to the ED when challenged with new healthcare issues or a perceived failure of current issues to resolve in a timely manner.

Daily alcohol use is associated with a *decrease* in risk of 30-day returns.^6^ However, two large retrospective cohort studies of older adults reported that a general history of substance abuse was an independent predictor of frequent ED use (five or more visits in one year).^24^, ^27^ Unfortunately, individual analyses for each of the substances of abuse that were included in these latter studies were not reported, making comparison of these disparate study conclusions difficult. Thus, it is unknown if daily alcohol use might confer a different risk compared to other substances of abuse.

### Health Literacy, Cognitive Health, and Physical Functioning

The Institute of Medicine defines health literacy as “the degree to which individuals can obtain, process, and understand basic health information and services they need to make appropriate health decisions.^29^ In older adults, low health literacy has been linked to decreased use of preventative services, higher utilization of acute care settings (such as the ED) and resources, and poorer health outcomes.^30^–^32^ Over 70% of elderly patients are not questioned on their ability to care for themselves prior to discharge; 20% disclose that they do not understand their discharge instructions.^33^, ^34^ This subset of the older adult population may have difficulty comprehending and following their discharge instructions. This may lead some patients to return when their initial complaints do not improve due to uncertainty and lack of comprehension regarding their discharge diagnoses, treatment, and follow-up plans.^22^

Several studies indicate poor cognitive health also is an important driver of ED returns.^6^, ^14^, ^15^, ^35^, ^36^ Older-adult patients with cognitive and memory impairment were at an increased risk for 30-day returns, and several studies demonstrated it to be an independent predictor for these returns.^6^, ^15^, ^37^ However, Ostir et al. found that poor cognitive health and odds of 30-day revisits did *not* have a significant association. Although, Ostir et al. did find that *higher* cognitive health scores were linked to *lower* risk for unplanned ED revisits at 60- and 90-days post-index visit. The authors found that every one-point increase in cognitive score was associated with 24% and 21% decreased odds of 60-day and 90-day revisits to the ED, respectively.

The lack of significant association between poor cognitive health and increased 30-day returns by Ostir et al. may be explained by several differences in the study population, which was mostly female (70.9%), African American (73.6%), and with cognitive impairment (94.7%). The average cognitive score of these patients was 4.5 points below standardized norms for persons 65 years and older,^35^ whereas 76.8% of the study population in the McCusker et al. study had no impairment or only mild cognitive impairment.^6^ Only 18.7% of patients in the de Gelder et al. study were found to have cognitive impairement.^16^ Since nearly all patients in the Ostir et al. study had cognitive impairment, their findings may be due to the lack of an adequate comparison group.

There are several possible explanations why patients with poor cognitive health may be at increased risk for recidivism, including suffering from more complex comorbidities necessitating more frequent healthcare, decreased comprehension of ED discharge diagnoses and instructions, and decreased accuracy in reporting of presenting illness. Patients with delirium superimposed on dementia were found to have lower concordance with their surrogates regarding reason for ED presentation reported to ED staff.^38^ This discordance between presenting complaints may lead to insufficient evaluation, missed diagnosis, and/or inappropriate discharge, particularly when the surrogate is not available during the ED evaluation.

In addition to cognitive health, poor physical function and poor general health also increase odds of returning within 30 days, and may be an independent predictor for ED recidivism.^6^, ^16^, ^26^ As physical functioning is a well-established predictor of outcomes among elderly patients, these findings likely reflect the characteristics of a sicker aging population.

### Patient Perceptions of ED Care

Several studies have shown that patients, despite access to care (insurance and a primary care physician), prefer to seek care in the ED compared to the outpatient setting. 39–41 Reasons include the following: accessibility/convenience; perceived urgency of complaints; inability to wait for scheduled primary care follow-up due to worsening of persistence of symptoms; expedited diagnostic testing; perceived availability of specialists; lack of transportation to primary care office; and wanting a second opinion, among other reasons. In a study of the general ED population, uninsured patients were not found to use the ED more than insured patients, but they use other types of care less. Interestingly, both the insured and uninsured visit the ED at similarly high rates for non-emergent complaints or complaints that can be treated in non-ED settings.^42^

As discussed previously, patient fear or uncertainty likely plays an important role in understanding why patients come (and return) to the ED. This sense of uncertainty regarding the cause of their symptoms is best illustrated by Castillo et al.’s findings of a rather high rate of older adults returning to the ED for the same primary diagnosis (23.2%) and many seeking care at a different facility (19.4%), perhaps in hopes of finding a different conclusion from their index ED visit.^27^ In a qualitative study of 40 adult patients with chronic cardiovascular disease or diabetes, patient reported driving factors for ED returns included feeling a sense of fear or uncertainty (rather than relief) with negative test results and expecting a diagnosis for their symptoms. Many patients who did not receive a clear diagnosis for their symptoms reported needing to return until a diagnosis was found.^20^, ^21^ In two studies of older adults, patients were less likely to consider that their complaint has been completely resolved and believed they would be less independent after discharge from the ED.^33^, ^43^

A survey of 15 older adults also linked patient perception of ED care with ED recidivism, including believing that the ED was their “only option” and that their symptoms required specialized care only provided in the ED.^22^ Several patients also reported that they believed their primary care physician would have advised them to seek care in the ED for their symptoms. Others reported receiving ineffective treatments or instructions at the time of ED discharge. In some cases, this perception may stem from inadequate patient counseling regarding expectations and reasonable goals of care and that can be achieved during the ED visit.

## FUTURE DIRECTIONS

The older adult population is a key and significant contributor to ED recidivism and is responsible for a disproportionate amount of healthcare costs. For this reason, older adults have received much attention and study to create interventions aimed at reducing ED recidivism. The unique characteristics of this patient group (complexity of medical issues, age and pathological-related changes in cognition and physical function) should be considered when developing strategies to minimize ED returns. The generation of a profile for elderly patients at increased risk for ED returns could identify potential targets for individualized education, counseling, and other interventions to reduce ED over-utilization.

Many of the study results discussed in this review were performed outside the U.S. and thus may not be fully generalizable to older adults residing in the U.S. due to different social and cultural influences and healthcare systems. However, when data was available for comparison, studies performed in the U.S. identified many similar risk factors for return visits in older adults as the non-U.S. studies. These similarities suggest that the underlying reasons for ED utilization by older adults may be influenced more by themes related to aging rather than the cultures or healthcare models of individual countries. However, it is important to note that these studies were all performed in highly developed countries with stable economies and well-established healthcare systems. Therefore, whether the identified risk factors would remain true in developing countries with fewer healthcare resources is unknown and deserves further study.

Numerous risk factors have been identified in the literature. Further study is needed to understand how each of these areas influences return visits, how they influence each other, and to resolve discrepancies in previously reported findings.

## Figures and Tables

**Table 1:** Characteristics of included studies.^4^–^6^, ^10^, ^14^–^18^, ^23^–^26^, ^27^, ^28^, ^35^–^37^, ^41^

Author (year)	Location	Study type	Study duration	Period of ED use	Sample size	Age of sample	Primary Outcome(s)
Hastings SN, et al. (2008)	U.S.	Retrospective review of Medicare Current Beneficiary Survey data	01/2000 to 09/2002	90 days	1851	65 years or older	ED return, hospital admission, nursing home admission or death
Hastings SN, et al. (2007)	U.S., VA medical center	Retrospective, cohort	07–09/2003	90 days	942	65 years or older	VA ED return, hospitalization, and/or death
McCusker J, et al. (2000)	Canada, 4 sites	Prospective observational cohort	1996 (3 month period)	6 months	1122	65 years or older	Early returns (within 30 days of index visit) and frequent returns (3 or more return visits in six months)
LaMantia MA, et al (2010)	U.S., 1 site	Retrospective review	2007 (1 year)	30 days	995	65 years or older	ED returns
de Gelder J, et al. (2018)	Netherlands, 3 sites	Prospective observational cohort	3 months in 2014 and 2015 for two sites. Third site not specified	30 days	1093	70 years or older	ED return and 90-day functional decline or mortality
McCusker J, et al. (1997)	Canada, 1 site	Prospective observational cohort	07–08/1994	90 days	167	75 years or older	ED returns
Southerland LT et al. (2016)	U.S, 1 site	Retrospective review	08/2011 to 02/2013	90 days	263	65 years or older	ED returns after discharge for fall from standing
Southerland LT et al. (2014)	U.S., 1 site	Retrospective review	08/2010 to 07/2011	72 hours	315	65 years or older	ED returns in patients with new fracture diagnosis
Howard R, et al. (2014)	Australia, 1 site	Prospective observational cohort	8 months	30 days	356	65 years or older	ED returns in patients discharged with pain
Brennan J, et al. (2017)	U.S.	Retrospective, review of non-public, visit-level data obtained from the California Office of Statewide Health Planning and Development	2013 to 2014 (2 years)	1 year	71,449	65 years or older	Frequent ED users (defined as 6 or more visits in one year)
Choi NG et al. (2012)	U.S.	Randomized control trial	2 years	6 months	121	50 years or older	Frequency of ED use
Friedmann PD, et al. (2001)	U.S/, 1 site	Prospective observational cohort	10/1995 to 06/1996	90 days	463	65 years or older	ED return, hospitalization, and/or death
Castillo EM, et al. (2017)	U.S., multicenter	Retrospective review	Not specified	7 days	871,558	65 years or older	ED returns
Naughton C, et al. (2010)	Ireland, 2 sites	Prospective observational cohort	18 months	6 months	306	65 years or older	ED returns
Ostir GV, et al. (2016)	U.S., 1 site	Prospective observational cohort	07–11/2014	90 days	110	65 years or older	ED returns and cognitive health
LaMantia MA, et al. (2016)	U.S.	Retrospective review of local electronic medical record data, Medicare claims, Indiana Medicaid claims, resident-level Minimum Data Set (MDS), and Outcome and Assessment Information Set (OASIS) data	11 years	30 days	32,697	65 years or older	ED use and returns
Lee J, et al. (2015)	Canada, 8 sites	Prospective observational study	04/2009 to 04/2013	6 months	1568	65–100 years of age	ED return or hospitalization after ED discharge with minor traumatic injury
Horney C, et al. (2012)	U.S, 1 site	Retrospective cohort	06–9/2007	90 days	308	65 years or older	Healthcare use including ED returns or hospitalizations

*ED*, emergency department; *U.S*., United States; *VA*, Veterans Administration.

**Table 2:** Reported frequency or odds of recidivism by diagnoses.^6^,^10^,^14^–^18^

Diagnoses*	All ED returns regardless of disposition %, OR (CI, p-value)	ED discharge with subsequent ED recidivism %, OR (CI, p-value)	Admission with subsequent ED recidivism OR (CI, p-value)	ED discharge with subsequent admission (%)
Circulatory system		142 (12.6%)		17 (3.6%)
Chest pain	343 (16.7%)		1.55 (1.14–2.12, 0.01)	
Foot/toe swelling		7.67 (1.78–33.04, 0.01)		
Hypertension	0.41 (0.16–1.02, 0.05)			
Respiratory system		81 (7.2%)		56 (12%)
Dyspnea	68 (6.2%)		1.73 (1.09–2.75, 0.02)	
General viral infection	9.37 (0.85–103.82, 0.07)			
Accidental injuries	463 (42.4%)	104 (9.2%); 1.48 (1.10–1.99, 0.01)		39 (8.3%)
Head trauma+	2.35 (1.06–5.2, 0.036)			
Leg/hip fracture	0.27 (0.06–1.11, 0.07)			
Fracture	1.24 (0.64–2.40, 0.518)			
Digestive		93 (8.2%)		35 (7.5%)
Abdominal pain	107 (5.2%)			
Stomach/abdominal pain	6.03 (1.34–27.12, 0.02)	5.72 (1.09–29.90, 0.04)		
Lower abdominal pain		4.18 (1.13–15.57, 0.03)		
Abdominal distention			12.23 (2.45–61.16, 0.00)	
Generalized weakness	141 (12.9%)		1.57 (1.06–2.32, 0.03)	
Disorders of speech/speech disturbance			5.67 (1.25–25.80, 0.03)	
Allergy, NOS		5.44 (1.33–22.28, 0.02)		
Epistaxis		3.39 (1.59–7.24, 0.00)		
Symptoms referable to the lips	10.26 (0.93–113.51)			
Urinary tract infection			3.00 (1.18–7.66, 0.02)	
Infection of skin of hand, arm, or finger		6.37 (1.17–34.66, 0.03)		

* Diagnoses and body systems follow the International Classification of Diseases, 9th Revision, classification system as reported in the cited literature.

+Derived from retrospective chart reviews of ED recidivism in patients after a fall.

*ED*, emergency department; *OR*, odds ratio; *CI*, confidence interval; *NOS*, not otherwise specified.
